# Phylogenetic analysis and complete chloroplast genome of *Salix wilhelmsiana* M.B.

**DOI:** 10.1080/23802359.2022.2119814

**Published:** 2022-09-15

**Authors:** Zhengxuan Wang, Huijie Tang, Zicheng Yu, Jing Wang, Xu Yao, Xiaoping Li

**Affiliations:** a Collaborative Innovation Center of Southern Modern Forestry, Nanjing Forestry University, Nanjing, China; b College of Forestry, Nanjing Forestry University, Nanjing, China; c Jiangsu Key Laboratory for Poplar Germplasm Enhancement and Variety Improvement, Nanjing Forestry University, Nanjing, China

**Keywords:** *Salix wilhelmsiana* M.B., chloroplast genome, phylogenetic analysis

## Abstract

*Salix wilhelmsiana* M.B. Bieberstein 1819 is a perennial woody plant with high economic and ecological value. In this study, we annotated the chloroplast (cp) genome of *Salix wilhelmsiana* M.B. The results showed that the length of the complete cp genome is 155,577 bp, which is typically composed of two single-copy regions (large single-copy (LSC) of 84,439 bp and small single-copy (SSC) of 16,221 bp) and a pair of IR regions of 27,457 bp with a quadripartite structure. The genome contains 129 genes, including 84 protein-coding genes, 37 tRNA genes, and eight rRNA genes. The GC content was 36.70%. Phylogenetic analysis based on cp genome sequences of 19 species from the Salicaceae family revealed that *S. wilhelmsiana* M.B. is closely related to *S. viminalis var. gmelinii.*

The *Salix* genus, comprised of trees known for their world-renowned landscape greening and economic value, contains approximately 400 natural species distributed worldwide (Zhou et al. 2021). There are approximately 200 species found across provinces in China. *Salix wilhelmsiana* M.B., one of the most important species of the *Salix* genus, is a shrub or small tree and is commonly used for its ornamental leaves. It is mostly grown in desert and semidesert areas in northern China and used for its drought-tolerant attributes. However, molecular breeding programs and germplasm resources for *S. wilhelmsiana* M.B. have been limited due to a lack of genome data. The objectives of our study were to sequence and to assemble the complete chloroplast (cp) genome of *S. wilhelmsiana* M.B. using next-generation sequencing. Having the complete cp genome of *S. wilhelmsiana* M.B. elucidated will provide data for determining the phylogeny of the *Salix* genus and advancing research on *S. wilhelmsiana* M.B.

Fresh leaves of *Salix wilhelmsiana* M.B. were collected in Yanchi County in the Ningxia Hui Autonomous Region (China, N37°46′55.82″, E107°24′9.33″). Leaf specimens (No. YCXYL2018003) were deposited in Room 60708 Biotechnology Building, Nanjing Forestry University, Nanjing, China (Li Xiaoping, xpli@njfu.edu.cn). Total genomic DNA was extracted using the improved CTAB method (Doyle and Doyle 1987). The Illumina HiSeq 2000 platform was used for sequencing after the sequencing library was constructed. Approximately, 6.1 GB of clean data were generated after filtering raw data with Fastp (Chen et al. 2018). Then, the clean data were used to assemble the complete cp genome using NOVOPlasty software version 4.1 (https://github.com/ndierckx/NOVOPlasty) (Dierckxsens et al. 2017). The complete cp genome of *Salix koriyanagi* was selected from the NCBI database as the reference sequence. CPGAVAS2 software was used for cp genome annotation combined with manual correction (Shi et al. 2019). The complete cp genome sequence of *S. wilhelmsiana* M.B. was submitted to GenBank with the accession number OL405086.

Like to most other angiosperms, the cp of *S. wilhelmsiana* M.B. is a typical quadripartite structure 155,577 bp in length, including a large single-copy (LSC) of 84,439 bp and a small single-copy (SSC) of 16,221 bp, reversed by two 27,457 bp inverted repeat sequences (IRs). The genome contains 129 genes, including 84 protein-coding genes, 37 tRNA genes, and eight rRNA genes. The total GC content of the circular DNA molecule was 36.70%. The GC content of the LSC was 34.43%, the SSC was 30.98%, and the IRs (IRA and IRB) were each 41.87% (Figure 1).

**Figure 1.**
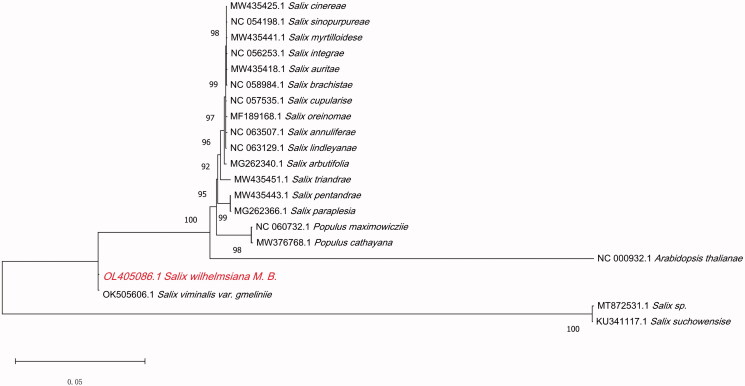
Phylogenetic relationships of *S. wilhelmsiana* M.B. and the other 19 species based on the chloroplast genome sequences, and *Arabidopsis thaliana* was used as an outgroup.

To reveal the relationship between *S. wilhelmsiana* M.B. and other species in the Salicaceae family, we conducted phylogenetic analyses. The cp genome sequences of 19 published Salicaceae species in the NCBI database, including seventeen *Salix* and two *Populus* species, were downloaded. *Arabidopsis thaliana* was used as an outgroup. All sequences were aligned using the program MAFFTv.7.149 (https://mafft.cbrc.jp/alignment/software/) (Katoh and Standley 2013), and a maximum-likelihood phylogenetic tree was constructed by MEGA 11 (Kumar et al. 2016) with 1000 bootstrap replicates. We used the Tamura–Nei model, the number of threads was 7, and we used a phylogram type of phylogenetic tree. The results showed that *Salix viminalis var. gmelinii* is more closely related and sister to a highly supported clade composed of 19 species (*Salix cinerea*, *Salix sinopurpurea*, *Salix myrtilloides*, *Salix integra*, *Salix aurita*, *Salix brachista*, *Salix cupularis*, *Salix oreinoma*, *Salix annulifera*, *Salix lindleyana*, *Salix arbutifolia*, *Salix triandra*, *Salix pentandra*, *Salix paraplesia*, *Populus maximowiczii*, *Populus cathayana*, *Salix viminalis var. gmelinii*, *Salix sp*., and *Salix suchowensis*). Our research lays a solid foundation for advanced studies on the genetic diversity and phylogeny of *S. wilhelmsiana* M.B.

## Data Availability

The complete chloroplast genome sequence data of *S. wilhelmsiana* M.B. supporting the findings of this study are openly available in GenBank of NCBI at http://www.ncbi.nlm.nih.gov/ under the accession number OL405086. The associated BioProject, SRA, and BioSample numbers are PRJNA778430, SRR16916512, and SAMN22959552, respectively.
